# Comparison of the Levels of Pro-Inflammatory Cytokines Released in the Vastus Lateralis Muscle of Patients with Fibromyalgia and Healthy Controls during Contractions of the Quadriceps Muscle – A Microdialysis Study

**DOI:** 10.1371/journal.pone.0143856

**Published:** 2015-12-01

**Authors:** Nikolaos Christidis, Bijar Ghafouri, Anette Larsson, Annie Palstam, Kaisa Mannerkorpi, Indre Bileviciute-Ljungar, Monika Löfgren, Jan Bjersing, Eva Kosek, Björn Gerdle, Malin Ernberg

**Affiliations:** 1 Section for Orofacial Pain and Jaw Function, Department of Dental Medicine, Karolinska Institutet, and the Scandinavian Center for Orofacial Neurosciences (SCON), Huddinge, Sweden; 2 Division of Community Medicine, Department of Medical and Health Sciences, Faculty of Health Sciences, Linköping University, Pain and Rehabilitation Center, Anaesthetics, Operations and Specialty Surgery Center, County Council of Östergötland, Linköping, Sweden; 3 Department of Rheumatology and Inflammation Research, Institute of Medicine, Sahlgrenska Academy, University of Gothenburg, Göteborg, Sweden; 4 Section of Health and Rehabilitation/Physiotherapy, Institute of Neuroscience and Physiology, Sahlgrenska Academy, University of Gothenburg, Göteborg, Sweden; 5 Centre for Person Centered Care (GPCC), University of Gothenburg, Göteborg, Sweden; 6 Department of Clinical Sciences, Karolinska Institutet, Danderyd Hospital, Stockholm, Sweden; 7 Department of Clinical Neuroscience, Karolinska Institutet, and Stockholm Spine Center, Stockholm, Sweden; University of Sevilla, SPAIN

## Abstract

**Objective:**

Fibromyalgia is associated with central hyperexcitability, but it is suggested that peripheral input is important to maintain central hyperexcitability. The primary aim was to investigate the levels of pro-inflammatory cytokines released in the vastus lateralis muscle during repetitive dynamic contractions of the quadriceps muscle in patients with fibromyalgia and healthy controls. Secondarily, to investigate if the levels of pro-inflammatory cytokines were correlated with pain or fatigue during these repetitive dynamic contractions.

**Material and Methods:**

32 women with fibromyalgia and 32 healthy women (controls) participated in a 4 hour microdialysis session, to sample IL-1β, IL-6, IL-8, and TNF from the most painful point of the vastus lateralis muscle before, during and after 20 minutes of repeated dynamic contractions. Pain (visual analogue scale; 0–100) and fatigue Borg’s Rating of Perceived Exertion Scale; 6–20) were assessed before and during the entire microdialysis session.

**Results:**

The repetitive dynamic contractions increased pain in the patients with fibromyalgia (P < .001) and induced fatigue in both groups (P < .001). Perceived fatigue was significantly higher among patients with fibromyalgia than controls (P < .001). The levels of IL-1β did not change during contractions in either group. The levels of TNF did not change during contractions in patients with fibromyalgia, but increased in controls (P < .001) and were significantly higher compared to patients with fibromyalgia (P = .033). The levels of IL-6 and IL-8 increased in both groups alike during and after contractions (P’s < .001). There were no correlations between pain or fatigue and cytokine levels after contractions.

**Conclusion:**

There were no differences between patients with fibromyalgia and controls in release of pro-inflammatory cytokines, and no correlations between levels of pro-inflammatory cytokines and pain or fatigue. Thus, this study indicates that IL-1β, IL-6, IL-8, and TNF do not seem to play an important role in maintenance of muscle pain in fibromyalgia.

## Introduction

Fibromyalgia (FM) is a musculoskeletal condition that is characterized by chronic widespread pain and tenderness (generalized allodynia/hyperalgesia) and often accompanied by fatigue, sleep disturbances, cognitive impairment and psychological distress [1–8]. FM has a prevalence of 2% to 4% in the general population and is more common in women than in men [2, 9–11]. FM is the second most common condition among the rheumatic disorders after osteoarthritis and is one of the most common conditions seen in the primary health care [12, 13]. It has a negative impact on the patients’ mental health, career and personal relationships [14]. Women with FM consistently score very low in quality of life (QoL) measures compared to other chronic conditions such as rheumatoid arthritis, osteoarthritis, and chronic obstructive pulmonary disease [15]. They also report difficulties with several daily life activities such as climbing stairs and walking two blocks [16].

The etiology of FM is still largely unknown. Previous studies have shown that patients with FM have an enhanced temporal summation [17], increased sensitivity to pressure [18], heat [18–20], cold [21] and electrical stimuli [22] as well as a prolonged decline of heat-induced pain in comparison to control subjects [17], consistent with central hyperexcitability. The impairments of descending pain modulation in patients with FM [19] are also reflected as aberrant cerebral pain processing [23]. Furthermore, when compared to controls patients with FM have reduced brain volumes [24] as well as reduced functional connectivity affecting brain regions associated with pain modulation [5]. However, it is suggested that peripheral input is important to maintain central hyperexcitability [25]. This abnormal pain processing, in combination with impaired endogenous analgesic systems [6] may indicate that FM is a multidimensional hyperalgesic pain syndrome where an altered central sensory processing interacts with peripheral pain input [25, 26].

A possible peripheral pathogenic factor in FM is an imbalance between pro- and anti-inflammatory cytokines, as this imbalance seems to play an important role in induction and maintenance of chronic pain [27]. Cytokines are small proteins that are mainly released from immune cells, but also from non-immune cells like fibroblasts and Schwann cells. They are classified as either pro- or anti-inflammatory. The pro-inflammatory cytokines are involved in painful chronic inflammatory diseases, while the anti-inflammatory cytokines have an analgesic effect [28]. Previous studies suggest that there is a dysfunction in the regulation of the release of the pro-inflammatory cytokines IL-1, IL-6 and IL-8 in patients with FM [29, 30] and that elevated levels of serum IL-8 are correlated with increased pain intensity in patients with FM [29]. Further, patients with FM have been reported to have increased levels of IL-8 both in serum and in cerebrospinal fluid compared to healthy controls [31–33] and it is suggested that higher levels of IL-6 and IL-8 are related to higher pain sensitivity and a disruption in the efficiency of the conditioned pain modulation [34]. However, this is not a general finding since one study indicates that the higher levels of IL-8 are not correlated with pain [33]. Several studies have shown an increased level in serum and/or plasma for IL-1, IL-6, IL-8 as well as TNF in patients with FM compared to healthy controls [29, 32, 35–40], although this is not a general finding [31].

Microdialysis can be used to analyze substances of interest in the interstitium of the muscle, as this method allows assessments of alterations of substances before they are diluted and cleared by the circulatory system. The extracellular matrix plays a key role in physiologic functions of cells, including the primary afferent nociceptor [41]. The muscles that have been analyzed with microdialysis are the masseter, trapezius, gastrocnemius and vastus lateralis [42]. This and other studies have shown that there is an increase of algogenic substances, such as glutamate, serotonin, substance P and calcitonin gene-related peptide in patients with chronic myalgia in the masseter or trapezius muscles [43–46]. Further, increased muscle levels of algogenic substances during low-force muscle-exercise have been reported in patients with chronic myalgia in the trapezius muscle [43, 45, 47]. Also, patients with FM have higher levels of serotonin and leukotriene B4 compared to patients with chronic localized myalgia of the temporomandibular system as well as healthy controls [48, 49]. It has further been reported that patients with FM have higher levels of pyruvate and lactate in the trapezius muscle compared to healthy controls [50, 51]. A few studies have shown that there are increased levels of cytokines in a myalgic trapezius muscle compared to a pain-free trapezius muscle [44, 46], however these studies had very small samples sizes. Also, other studies have shown increased release of interstitial levels of IL-6 during repetitive, low-force exercise in the myalgic trapezius muscle of patients with chronical myalgia of the trapezius muscle and whiplash associated disorders [52, 53]. Yet, no studies have until now investigated the levels of IL-6 and other pro-inflammatory cytokines during repeated dynamic contractions of the vastus lateralis in patients with FM. Taken together, little is known regarding potential peripheral mechanisms, such as the release of pain mediators [54], as a contributing factor in the maintenance of FM.

Based on previous research we hypothesized that there are differences in the levels of pro-inflammatory cytokines in patients with FM compared to healthy controls [32, 55] and that there is a correlation between the level of pro-inflammatory cytokines and the level of induced pain as well as fatigue during a low-force muscle exercise. Hence, the primary aim of this study was to investigate the levels of pro-inflammatory cytokines released in the vastus lateralis muscle during repetitive dynamic contractions of the quadriceps muscle in patients with FM and healthy controls. As a secondary aim, this study investigated if the levels of pro-inflammatory cytokines were correlated with pain or fatigue during these repetitive dynamic contractions.

## Materials and Methods

This multicenter study was carried out during the period of 2010–11 to 2013–05 as a part of a randomized controlled trial regarding the effects of strength and relaxation exercise on FM symptoms (clinicaltrials.gov (NCT01226784)) in the three participating sites Stockholm, Linköping and Gothenburg [56]. The present study was carried out in selected centers in each site and the centers were the Rehabilitation Medicine, Department of Medical and Health Sciences (IMH), Faculty of Health Sciences, Linköping University, and the Pain- and Rehabilitation Centre, County Council of Östergötland, both in Linköping; the Department of Rheumatology and Inflammation Research, Institute of Medicine, Sahlgrenska Academy, University of Gothenburg, Göteborg; and the Department of Clinical Sciences, Karolinska Institutet at Danderyd University Hospital, Stockholm, all in Sweden. The methods and selection of participants were approved by the regional ethical review board in Stockholm, Sweden (2010/1121-31/3). All participants were above 18 years of age and the study followed the principles for medical research according to the guidelines of the Declaration of Helsinki as well as the International Conference on Harmonisation Guideline for Good Clinical Practice. All participants received both written as well as verbal information and gave their verbal and written consent.

### Participants

#### Patients with fibromyalgia

The study comprised thirty-two female patients with a clinical diagnosis of FM. They formed a subgroup out of 130 patients that participated in the randomized controlled trial regarding the effects of strength and relaxation exercise on FM symptoms [56]. Their background data are presented in Table 1. The participants were recruited through advertisements in the local newspapers of the three participating cities (Gothenburg, Stockholm, and Linköping).

**Table 1:** The distribution of the age-matched participants, divided by 32 patients with fibromyalgia and 32 healthy controls. Their mean (SD) age (years), mean (SD) BMI (kg/m^2^), median (IQR) duration of pain (years), mean (SD) scores of depression, anxiety and quality of life, and median (IQR) intensity of pain (VAS), fatigue (Borg’s RPE) before microdialysis (-5 min) and at baseline (i.e. before the dynamic contractions at 120 min) is shown.

Participants	Fibromyalgia	Controls	P-values
**Age**	53.4 (8.6)	53.7 (9.4)	ns
**BMI**	27.9 (5.7)	24.6 (4.4)	.007
**Duration of pain**	13.4 (8.4)	-	ns
**Intensity of pain before microdialysis**	40 (37.5)	0 (0)	< .001
**Intensity of pain at baseline**	47.5 (30)	0 (0)	< .001
**Fatigue before microdialysis**	6 (1)	6 (0)	ns
**Fatigue at baseline**	6 (1)	6 (0)	ns
**HAD-Depression**	6.1 (4.0)	1.3 (2.9)	< .001
**HAD-Anxiety**	6.7 (4.2)	3.3 (3.4)	< .001
**SF36-PSC**	30.6 (7.7)	55.3 (4.1)	< .001
**SF36-MSC**	42.1 (11.5)	51.9 (5.9)	< .001

SD = Standard deviation; BMI = body mass index; IQR = Interquartile range (75 percentile minus 25 percentile); VAS = Visual analogue scale (0–100); Borg’s RPE = Borg’s Rating of Perceived Exertion Scale (6–20); HAD-Depression = Hospital Anxiety and Depression Scale–subscale depression; HAD-Anxiety = Hospital Anxiety and Depression Scale–subscale anxiety; SF36-PCS = Short Form Health Survey–subscale physical summary components; SF36-MSC = Short Form Health Survey–subscale mental summary components.

The inclusion criteria were age between 20 and 65 years, female sex and a diagnosis of FM according to the fibromyalgia classification criteria of the American College of Rheumatology (ACR) 1990 [1]. Exclusion criteria were high blood pressure (>160/90 mmHg), osteoarthritis (OA) in hip or knee, other severe somatic or psychiatric disorders, other dominating causes of pain than FM, high consumption of alcohol, participation in a rehabilitation program within the past year, regular resistance exercise or relaxation exercise twice a week or more, inability to understand or speak Swedish, and not being able to refrain from analgesics, NSAID or hypnotics for 48 hours prior to examination [56]. Use of NSAID for one week prior to microdialysis session was an additional exclusion criterion.

#### Healthy controls

Thirty-two age-matched female controls (CTR) with a mean (SD) age of 53.7 (±9.4) years participated, coinciding the mean age of the patients with FM, Table 1. They were also recruited through advertisements in the local newspapers of the three participating cities (Gothenburg, Stockholm, and Linköping) and formed a subgroup of the 42 controls that participated in the above mentioned trial. The inclusion criteria were age 20 and 65 years, and female sex. Exclusion criteria were any pain condition, high blood pressure (>160/90 mmHg), osteoarthritis (OA) in hip or knee, other severe somatic or psychiatric disorders, high consumption of alcohol, participation in a rehabilitation program within the past year, regular resistance exercise or relaxation exercise twice a week or more, inability to understand or speak Swedish, and not being able to refrain from analgesics, NSAID or hypnotics for 48 hours prior to examination.

### Experimental protocol

**Fig 1:**
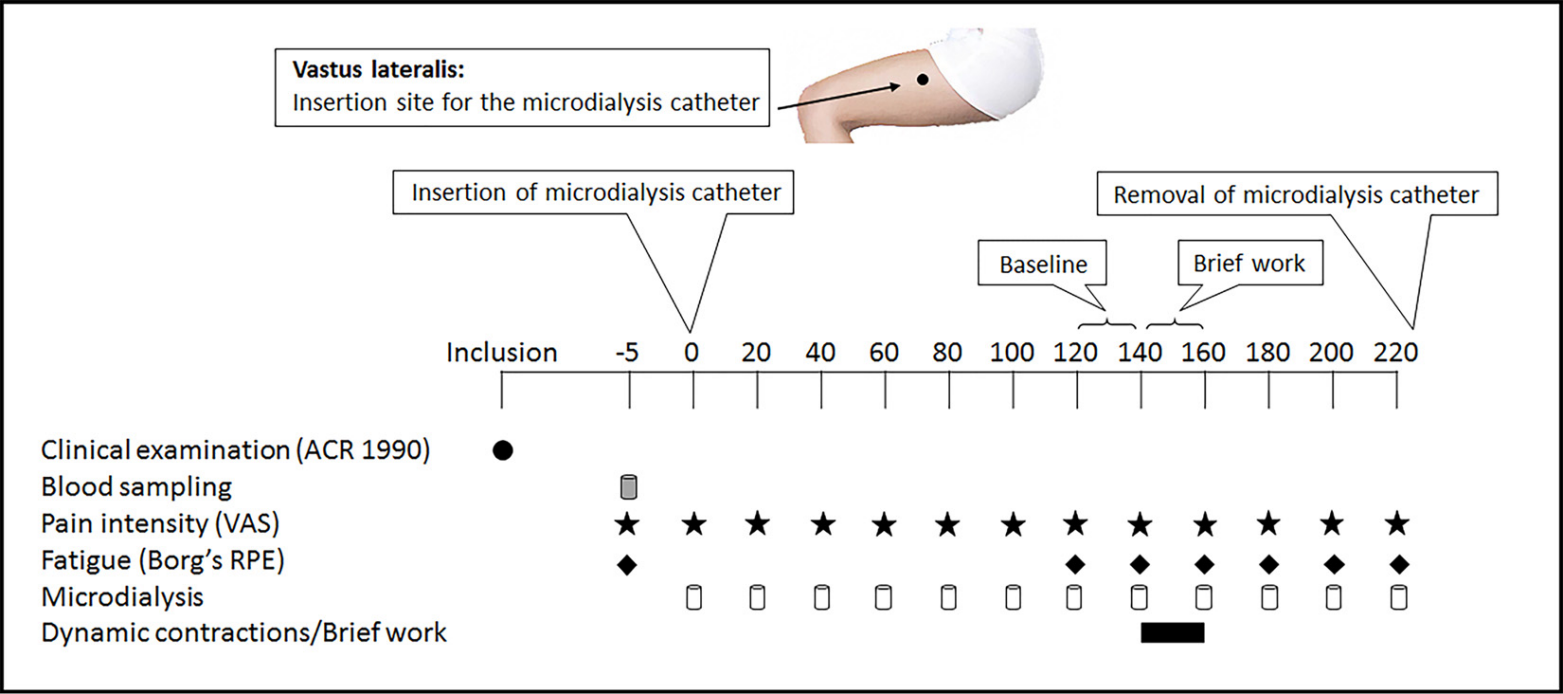
Schematic illustration of the study design. The flow-chart shows the time-points (min) for the blood sample, assessments of pain intensity, fatigue, microdialysis and dialysate sampling as well as the brief work (consisting of repetitive dynamic contractions of the quadriceps muscle) in 32 female patients with fibromyalgia and 32 healthy, pain-free age-matched controls. The pain intensity was assessed on a visual analogue scale (VAS; 0–100) while fatigue was assessed with Borg’s Rating of Perceived Exertion (RPE) Scale before, during and after the brief work.

This study comprised a single microdialysis session that lasted four hours. The microdialysis was performed in the most painful vastus lateralis muscle in order to sample the pro-inflammatory cytokines IL-1β, IL-6, IL-8 as well as TNF. After the 2 hours of stabilization, i.e. the trauma-phase, baseline dialysates were sampled between 120–140 min. This was followed by sampling of dialysates during the 20 min of standardized repeated dynamic contractions of the quadriceps muscle (brief work) and finally during the 60 min of recovery period. The brief task was to extend the knee 15° by slowly lifting the leg (resting with the knee bent 15° on a Pilatus ball; 55 cm diameter) to a straight position (0°) and then slowly lowering it down on the ball for participants with a current pain intensity in the exercising leg greater than 40 on a 0–100 visual analogue scale (VAS). For the other participants (current pain intensity less than 40 on a 0–100 VAS) the knee extension was 20°. Each repetition lasted for 5 sec, without any rest between repetitions. When the microdialysis was finished the catheter was removed and the membrane was checked to make sure that no damage had occurred. Prior to the experiment a venous blood sample was taken in order to analyze plasma levels of the pro-inflammatory cytokines, Fig 1.

### Pain intensity and fatigue

Pain intensity (visual analogue scale; VAS; 0–100) and fatigue (Borg’s Rating of Perceived Exertion (RPE) Scale; 6–20 [57]) were assessed before insertion of the microdialysis catheter (-5 min) and every five min during the dynamic contractions (140–160 min). Further, VAS was assessed immediately after insertion and then every 20 minutes, when the dialysates were changed, Fig 1.

### Assessment of Anxiety, Depression and Quality of life

The validated Swedish versions of the Hospital Anxiety and Depression Scale (HADS) were completed by all participants in order to assess anxiety and depression [58, 59], and the Short Form Health Survey (SF36) to assess quality of life [60]. From the SF36 the physical (SF36-PSC) and mental (SF36-MSC) summary components were calculated and used in the study. For a complete description of these questionnaires, see Sullivan and co-workers (1998) as well as Persson and co-workers (1998) [61, 62].

### Blood sampling

The venous blood samples were collected before the microdialysis with a Vacutainer (BD Vacutainer® Eclipse™ Blood Collection Needle, BD Diagnostics, Becton, Dickinson and Company, New Jersey, USA) in a 10 mL plasma tube (BD Vacutainer® Plus Plastic K_2_EDTA Tubes BD Diagnostics, Becton, Dickinson and Company, New Jersey, USA). The blood samples were centrifuged for 30 min (1500 g, +4°C) immediately after the collection, and the plasma was pipetted to Eppendorf tubes, aliquoted and stored at -70°C. The plasma levels of the pro-inflammatory cytokines IL-1β, IL-6, IL-8, and TNF were later analyzed to determine if the muscle levels obtained from the dialysates were locally produced or emanated from the blood plasma.

### Microdialysis of the vastus lateralis muscle

In order to sample the peripheral pro-inflammatory cytokines IL-1β, IL-6, IL-8, and TNF in the vastus lateralis muscle microdialysis was performed. After identification of the insertion point which was the belly of the vastus lateralis, at half the distance between the trochanter and the knee, (Fig 1) the skin overlying was anaesthetized with local anesthesia (Xylocain®, lidocaine 10 mg/mL, AstraZeneca, Södertälje, Sweden) five minutes prior to the insertion. Two commercially available microdialysis catheters (cut-off points of 100 kDa (CMA 71) and 20 kDa (CMA 60), CMA Microdialysis AB, Solna, Sweden; membrane 30-mm length, 0.5-mm diameter) were inserted into the vastus lateralis collateral to the muscle fibers at an angle of 45° to its full length. To determine the concentrations of IL-1β, IL-6, IL-8, and TNF the catheter with the 100 kDa (CMA 71) was used for the present study. The concentrations of small molecules such as lactate, pyruvate, glutamate, and glucose will be presented in another study. During the insertion of the catheter the typical slight resistance and involuntary muscle contraction was felt when penetrating the muscle fascia. In order to allow the tissue to recover from possible changes induced in the interstitial environment the participants were instructed to relax in a comfortable position on a bed during the trauma phase immediately after insertion of the catheter, i.e. the first 120 min.

The microdialysis catheter was perfused at a rate of 5 μL/min with a 2-mL syringe connected to a high-precision micro-infusion pump (CMA 107, CMA Microdialysis AB, Kista, Sweden). The perfusion medium consisted of a Ringer-acetate solution (Baxter Viaflo, Baxter Medical AB, Kista, Sweden) containing 0.5 mM Ringer-lactate (Baxter Viaflo, Baxter Medical AB, Kista, Sweden).

Microdialysates (100 μL) were collected every 20 min in 200-μL microvials, specially designed to collect micro-volume samples and minimize evaporation (CMA Microdialysis AB, Kista, Sweden), immediately pipetted to Eppendorf tubes and stored at -70°C. Only the dialysates collected at baseline (120–140 min), during the repeated dynamic contractions (brief work at 140–160 min), and during recovery period (160–220 min) were used for the analysis of the pro-inflammatory cytokines (Fig 1).

### Analyses of plasma and dialysates

The plasma and dialysate samples were analyzed at the Clinical Research Department, Department of Dental Medicine, Karolinska Institutet, Huddinge, Sweden with the commercial High Sensitivity Human Cytokine Magnetic Bead Panel Immunoassay (MILLIPLEX®MAP for Luminex® xMAP® Technology, EMD Millipore, Missouri, USA). The minimum detectable concentration (MinDC) were 0.06 pg/mL for IL-1β, 0.20 pg/mL for IL-6, 0.05 pg/mL for IL-8, and 0.07 pg/mL for TNF. Samples that were regarded as “out of range” by the program were considered as 0 pg/mL.

### Statistics

The SigmaPlot for Windows, version 13.0 (Systat Software Inc., Chicago, IL, USA) and SIMCA-P+ version 13.0 (Umetrics Inc., Umeå, Sweden) were used to analyze the data. For all tests the level of significance was set to *P* < .05. Mean and SD were used for descriptive statistics regarding age, while median and interquartile range (IQR) were used for descriptive statistics regarding pain intensity and fatigue. The Shapiro-Wilk test assessed data for normality. Non-parametric statistics were used to analyze the substances since they were not normally distributed and attempts to transform data did not change this. Friedman two-way repeated measures analysis of variance (RM ANOVA) was used to analyze changes in interstitial levels of pro-inflammatory cytokines, pain levels and fatigue over time. Holm-Sidak correction was used as post-hoc test when the Friedman test indicated a significant difference. To analyze differences between patients with FM and CTR at the different time points, the Mann-Whitney *U*-test, paired *t*-test or χ^2^-test were used.

Spearman’s correlation test, adjusted for multiple testing with Bonferroni correction, was used for analyses of significant correlations between pain intensity and/or fatigue induced by repetitive dynamic contractions and the release of biomarkers at 160 minutes (the first dialysate sample after the dynamic contractions).

Traditional statistical methods quantify the level of individual metabolites but disregard inter-relationships between different metabolites [63]. Thus, these methods assume variable independence when interpreting the results [64]. In order to investigate these relations multivariate data analyses (MVDA) were used. MVDA are capable of handling a number of inter-correlated substances and uses both advanced Principal Component Analyses (PCA) and Partial Least Squares (PLS) regressions as important tools. When investigating the multivariate correlations between the release of pro-inflammatory cytokines in dialysate and the level of the pro-inflammatory cytokines in blood as well as any correlation to pain, fatigue and group membership (i.e. FM or CTR) PLS were applied using SIMCA-P+ [65]. Before this analysis PCA was used to check for multivariate outliers. In the PLS analyses, AUC of the different biochemical substances were used.

## Results

### Baseline data

Table 1 shows background data and baseline values for pain and fatigue for the two groups as well as the levels of depression, anxiety and quality of life. The patients with FM reported significantly higher pain intensity compared to the CTR both before the microdialysis (-5 min) and at baseline (120 min). They also had significantly higher body mass index (BMI), and higher HAD-scores for depression and anxiety. Both quality of life indices were noticeable lower in patients with FM. However, the HAD-scores were within the normal range in both groups. No other differences were reported.

### Pain intensity and fatigue throughout the microdialysis experiment

#### Pain intensity

**Fig 2:**
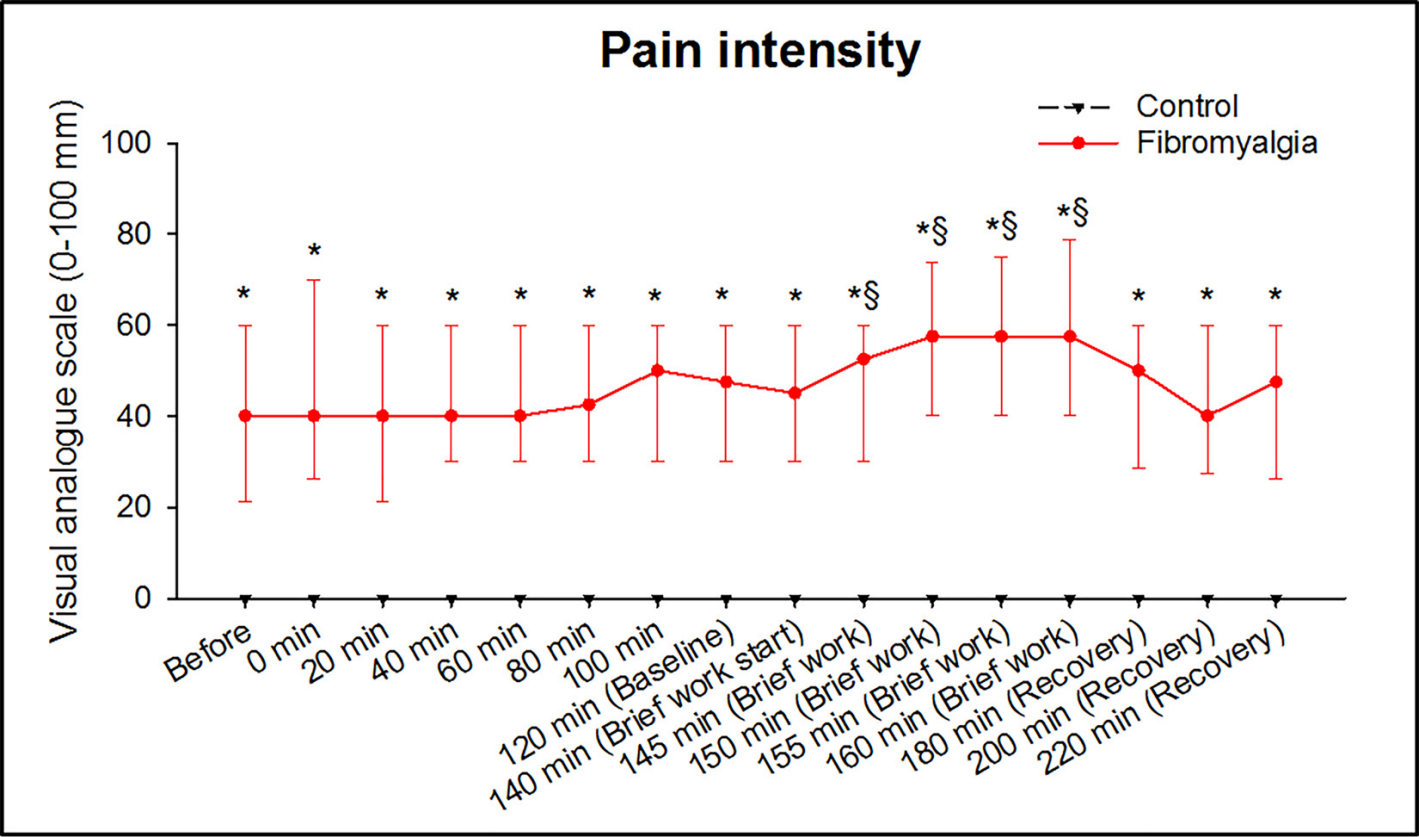
The pain intensity increased significantly only in patients with fibromyalgia (FM) by the brief work. Median (IQR) pain intensity in 32 women with FM and 32 healthy, pain-free age-matched controls (CTR), before and during repeated dynamic contractions of the quadriceps muscle (brief work). The pain induced by the catheter and the insertion of the catheter in FM decreased to the initial level, at baseline before the brief work. * = The brief work increased the pain intensity significantly in FM but not in the CTR (Friedman ANOVA; P < .001). After the brief work the pain intensity decreased to the initial level for FM. § = The pain intensity was significantly higher in FM at all time-points during the brief work compared to CTR (Holm-Sidak; P < .001,).

The catheter and the insertion of the catheter increased the pain intensity in 11 out of 32 patients with FM but did not induce any pain in the CTR (Fig 2). At baseline (120 min) the pain intensity had decreased to the initial level, Fig 2.

The pain intensity level remained at the initial level during the trauma phase in both groups. The dynamic contractions increased the pain intensity significantly in the patients with FM but not in the CTR (*P* < .001, Friedman ANOVA). After the brief work (consisting of repeated dynamic contractions) the pain intensity decreased to the initial level for the patients with FM. Further, the post-hoc test showed that the pain intensity was significantly higher in the patients with FM than CTR at all time-points during the dynamic contractions (*P* < .001, Holm-Sidak), Fig 2.

#### Fatigue

**Fig 3:**
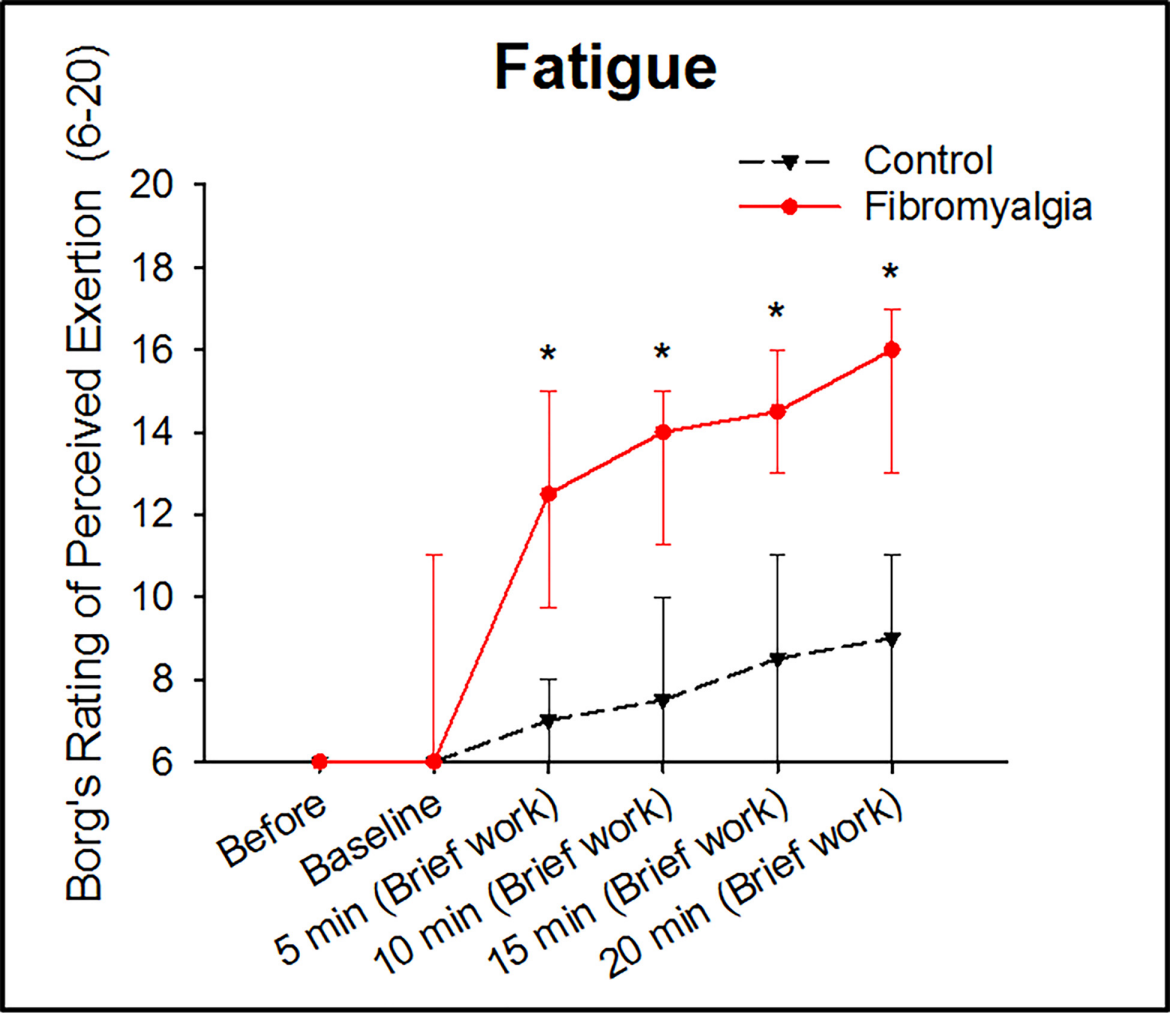
Brief work induced fatigue in both groups, but significantly higher in patients with fibromyalgia (FM). Median (IQR) perceived fatigue by 32 women with FM and 32 healthy, pain-free age-matched controls, before and during repeated dynamic contractions of the quadriceps muscle (brief work). * = The brief work induced fatigue in both groups (Friedman ANOVA: P < .001). The perceived fatigue was significantly higher at all time-points during the brief work in patients with fibromyalgia (Holm-Sidak; P < .001).

Perceived fatigue was similar between groups before the experiment and increased significantly in both groups during the brief work both over time and compared to baseline (120 min) (*P* < .001, Friedman ANOVA). Further, the post-hoc test showed that the perceived fatigue was significantly higher in the patients with FM at all time-points during brief work (*P* < .001, Holm-Sidak), Fig 3.

### Release of pro-inflammatory cytokines

**Fig 4:**
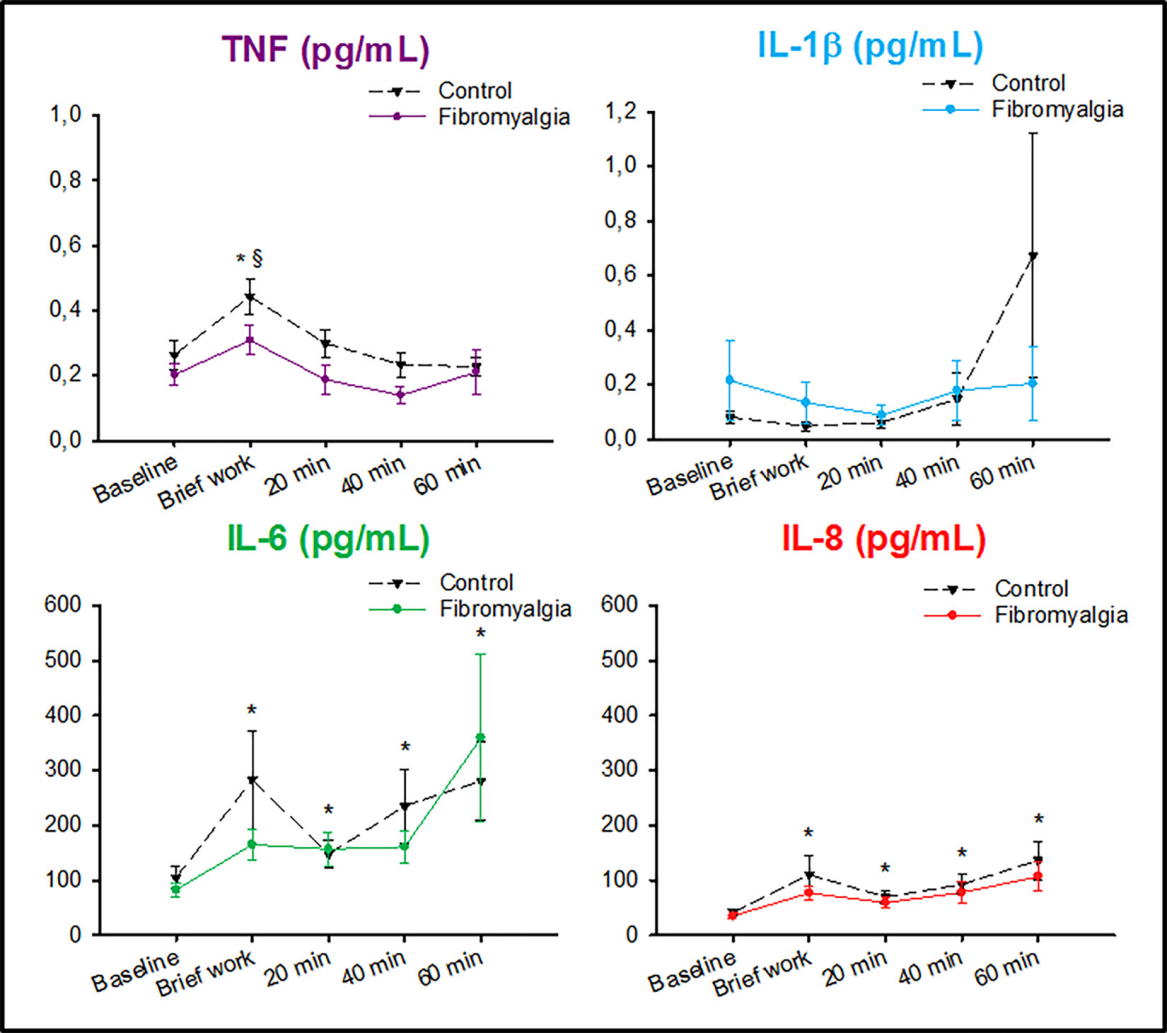
IL-6 and IL-8 increased significantly during and after the brief work in both groups. Mean (SEM) interstitial levels of the pro-inflammatory cytokines IL-1β, IL-6, IL-8, and TNF in 32 women with fibromyalgia (FM) and 32 healthy, pain-free age-matched controls (CTR), before and during repeated dynamic contractions of the quadriceps muscle (brief work). The levels of IL-1β did not change in either group. * = The levels of TNF did not change in FM, but increased in CTR. The levels of IL-6 and IL-8 did not differ between groups, but increased during and after the brief work in both groups (Friedman ANOVA: P < .001). § = The levels of TNF were significantly higher in CTR than in FM (Holm-Sidak: P = .033).

IL-1β was detectable in 48% of the dialysates in patients with FM, and 41% in CTR. The levels of IL-1β did not change during the brief work in either group and there were no differences between FM and CTR, Fig 4.

Both IL-6 and IL-8 were detectable in 90% of the dialysates in patients with FM and 97% in CTR, respectively. The levels of IL-6 and IL-8 increased significantly in both patients with FM and in CTR during the brief work period (*P* < .001, Friedman ANOVA). However, there were no differences in the levels of IL-6 and Il-8 between FM and CTR at any time-point, Fig 4.

TNF was detectable in 67% of the dialysates in patients with FM patients, which was significantly less than the 93% in CTR (*P* < .001, χ^2^-test). The levels of TNF did not change during the brief work in patients with FM, but increased significantly in CTR (*P* < .001, Friedman ANOVA). The post-hoc test revealed that the levels of TNF in CTR were significantly higher than in patients with FM during the brief work (*P* = .033, Holm-Sidak), Fig 4.

In blood plasma there were no significant group differences in the levels of any of the pro-inflammatory cytokines. The median (IQR) levels of IL-1 β in blood was 1.1 (2.2) pg/mL in patients with FM and 1.2 (1.5) pg/mL in CTR. The corresponding values for; **a)** IL-6 was 1.5 (1.4) pg/mL in FM and 1.3 (1.3) pg/mL in CTR; **b)** IL-8 was 1.5 (2.1) pg/mL in FM and 0.5 (2.3) pg/mL in CTR; and **c)** TNF was 4.2 (1.7) pg/mL in FM and 3.9 (3.7) pg/mL in CTR.

The multivariate regression analysis could not reveal any significant differences regarding the release of any of the cytokines, in the dialysates, between the FM patients and CTR. Neither did the multivariate regression analysis show any significant differences between CTR and FM patients regarding the levels of pro-inflammatory cytokines in blood.

### Correlations between pain, fatigue and pro-inflammatory cytokines

No correlations between pain intensity and the level of pro-inflammatory cytokines, nor between fatigue and the level of pro-inflammatory cytokines were revealed in the statistical analysis.

## Discussion

The main findings of this study were that the release of IL-6 and IL-8 during the brief work consisting of repeated dynamic contractions was similar in both patients with FM and age- and sex-matched controls. However, the release of TNF was lower in FM and did not increase during the dynamic contractions in contrast to CTR. Further, no correlations between the levels of pro-inflammatory cytokines and pain or fatigue were found.

This study showed an increase of the pro-inflammatory cytokine IL-8 and an increased pain intensity during the brief work. This is partly is in line with previous studies showing that blood serum levels of IL-8 are positively correlated to pain characteristics in post-menopausal patients with FM [66], and that increases in pain scores are positively correlated to increases in IL-8 [29, 39, 67]. Further, plasma levels of IL-8 were reported to be elevated after injections of lipopolysaccharide, as an experimental model of systemic inflammation and in serum during moderate exercise [55]. This increase of IL-8 was related to higher pain sensitivity and a disruption in the efficiency of the conditioned pain modulation in women [34]. Finally, it has been reported that patients with FM have increased levels of IL-8, compared to healthy controls, both in serum and in cerebrospinal fluid [31]. Taken together this indicates that IL-8 might play a contributing role in the development of chronic pain in patients with FM. In contrast to the present study, other studies indicate that the levels of IL-8 are higher in patients with FM compared to CTR [33, 55] without any correlations to pain [33]. Taken together, IL-8 seem to contribute to development or maintenance of muscle pain but not specifically in FM when compared to CTR. Hence any conclusion regarding this must be considered cautiously since these results are contradictory.

Also IL-1β, IL-6, and TNF seem to be highly important in the development of chronic pain since they contribute to spontaneous nociceptor activity triggering the development of chronic/persistent pain such as FM [53, 68]. It has moreover been shown in animal studies that they contribute to the induction of allodynia and hyperalgesia, commonly found in patients with FM [69, 70]. Further, the release of IL-1β, IL-6, and TNF during moderate exercise has also been shown to be higher in patients with fibromyalgia than healthy controls [55]. Previous studies that have shown that pro-inflammatory cytokines play an important role in both experimental [71] and chronic/persistent pain conditions since they act directly on nociceptive terminals that innervate inflamed tissues [72, 73]. The results from this study did not show any difference between patients with FM and CTR neither in the dialysate nor in blood, except for lower levels of TNF in patients with FM both at baseline and during the brief work, which is in contrast to the previous study where the release of TNF was higher in patients with FM [55]. It was recently suggested that TNF has a central role in adult myogenesis and in maintenance of muscle homeostasis [74], and that it promotes muscle regeneration via expansion of muscle stem cells [75]. TNF is expressed in myoblasts and a rapid increase of TNF expression takes place during the early hours of differentiation [76]. Thus, one could speculate that the release of TNF is blunted in skeletal muscles of patients with FM due to sustained muscle activity (between muscle contractions) leading to or reflecting an impaired muscle regeneration, and that this ultimately may reduce muscle tissue quality. However, the results from this study are in line with some recent studies indicating that there is no difference between FM and CTR regarding the blood levels of IL-1β, IL-6, and TNF [33, 66, 77], but contradictory to other studies reporting both lower [31] and higher [39] levels of TNF in patients with FM compared to healthy CTR. Altogether, the results from this study indicate that the pro-inflammatory cytokines IL-1β, IL-6, and TNF do not seem to play an important role in maintenance of muscle pain in patients with FM.

As expected, the patients with FM showed an increased pain intensity during the brief work (repeated dynamic contractions), which was not found in the CTR, and a significantly higher degree of fatigue compared to CTR during the exercise. These findings coincide with the results from previous studies and are among the characteristics of patients with FM [3, 8, 78]. Previous studies have described that fatigue in FM has several dimensions. The peripheral or physical fatigue is dependent on muscle contractions, and this is in agreement with the results from the present study that showed an increase in fatigue during dynamic contractions [79, 80], while central or mental fatigue mainly is associated with cognitive impairment [79]. Previous studies have also reported a positive correlation between pain and fatigue [81], and that increased ratings of fatigue are highly correlated to increased ratings of pain intensity at exhaustion, indicating that patients with FM have a more pronounced response to the input from the muscle nociceptors than healthy CTR [78]. Although pain and fatigue are strongly associated with depression, anxiety and sleep quality in patients with FM this study indicates that physical activity rather than pain induces increase of fatigue in FM. This is in accordance with a recent study indicating that pain is not the main driver for fatigue but a contributing factor [27].

One strength of this study is the relatively large number of participants in each group, compared to other microdialysis studies comprising patients with FM [50, 82]. Another strength is the well-established microdialysis technique which is a valuable tool to collect cytokines in human skeletal muscle during exercise [83] since the recent larger-pore membranes allow recovery of large molecules [84].

There are some limitations of this study that need to be addressed. One limitation of this study could be related the catheter itself since it might cause cell damage with localized bleeding, disturbing the tissue homeostasis for up to 7 hours after catheter insertion leading to a cytokine response to the catheter insertion [85]. One early study indicates that there was a higher release of serotonin in patients with FM than controls during the trauma phase [48], however it is unclear if there is a difference in the release of cytokines during the trauma phase between patients with FM and healthy controls. Nevertheless, this does not seem to have affected the results since there were no differences in cytokine level between the groups at the baseline registration. The present study did not investigate the release of anti-inflammatory cytokines which might be considered another limitation since lower blood mRNA levels of IL-10 have been reported in patients with FM [27] as well as a blunted response to exhaustive exercise [86], indicating a decreased anti-inflammatory cytokine activity in patients with FM. Therefore, further microdialysis studies that include both pro- and anti-inflammatory cytokines are warranted. There are some studies indicating that depression may be associated with elevated levels of IL-1β, TNF, and IL-6 [87, 88]. Thus, depression may be a confounding factor regarding cytokine levels in FM. In this study patients with severe psychiatric disorders were excluded. Moreover, the levels of depression and anxiety were assessed and found to be within the normal range both in the patients FM and controls [59]. Therefore, the levels of depression and anxiety probably did not have any major influence on the results. In addition, there were no differences in cytokine levels between groups. This also brings up the generalizability of the results. Since the participants were recruited to an exercise study they probably had less severe disease than FM-patients in general. On the other hand, studies of FM-populations are mostly conducted at tertiary care clinics, where the most severe cases are found. Nevertheless, inclusion of patients with more severe FM might have led to different results. Future studies may address this issue.

In conclusion, there were no differences between FM and CTR in the release of pro-inflammatory cytokines, and there were no correlations between pain or fatigue and release of pro-inflammatory cytokines. Thus, this study indicates that IL-1β, IL-6, IL-8, and TNF released in the interstitium of the muscle or in blood do not play an important role in maintenance of chronic muscle pain and fatigue in FM patients.

## References

[1] Wolfe F, Smythe HA, Yunus MB, Bennett RM, Bombardier C, Goldenberg DL, et al The American College of Rheumatology 1990 Criteria for the Classification of Fibromyalgia. Report of the Multicenter Criteria Committee. Arthritis and rheumatism. 1990;33(2):160–72. . 2306288 10.1002/art.1780330203

[2] Wolfe F, Ross K, Anderson J, Russell IJ, Hebert L. The prevalence and characteristics of fibromyalgia in the general population. Arthritis and rheumatism. 1995;38(1):19–28. . 7818567 10.1002/art.1780380104

[3] Wolfe F, Clauw DJ, Fitzcharles MA, Goldenberg DL, Katz RS, Mease P, et al The American College of Rheumatology preliminary diagnostic criteria for fibromyalgia and measurement of symptom severity. Arthritis care & research. 2010;62(5):600–10. 10.1002/acr.20140 . 20461783

[4] Jin H, Patil PM, Sharma A. Topical review: the enigma of fibromyalgia. Journal of oral & facial pain and headache. 2014;28(2):107–18. 10.11607/ofph.1220 . 24822234

[5] Flodin PD, Martinsen S, Lofgren M, Bileviciute-Ljungar I, Kosek E, Fransson P. Fibromyalgia is associated with decreased connectivity between pain- and sensorimotor brain areas. Brain connectivity. 2014 10.1089/brain.2014.0274 . 24998297 PMC4202907

[6] Smith HS, Barkin RL. Fibromyalgia syndrome: a discussion of the syndrome and pharmacotherapy. Disease-a-month: DM. 2011;57(5):248–85. 10.1016/j.disamonth.2011.02.001 . 21628007

[7] Smith HS, Harris R, Clauw D. Fibromyalgia: an afferent processing disorder leading to a complex pain generalized syndrome. Pain physician. 2011;14(2):E217–45. . 21412381

[8] Mease P. Fibromyalgia syndrome: review of clinical presentation, pathogenesis, outcome measures, and treatment. The Journal of rheumatology Supplement. 2005;75:6–21. . 16078356

[9] Miedema HS, van der Linden SM, Rasker JJ, Valkenburg HA. National database of patients visiting rheumatologists in The Netherlands: the standard diagnosis register of rheumatic diseases. A report and preliminary analysis. British journal of rheumatology. 1998;37(5):555–61. . 9651085 10.1093/rheumatology/37.5.555

[10] Henriksson CM, Liedberg GM, Gerdle B. Women with fibromyalgia: work and rehabilitation. Disability and rehabilitation. 2005;27(12):685–94. 10.1080/09638280400009089 . 16012061

[11] McBeth J, Jones K. Epidemiology of chronic musculoskeletal pain. Best practice & research Clinical rheumatology. 2007;21(3):403–25. 10.1016/j.berh.2007.03.003 . 17602991

[12] Hawkins RA. Fibromyalgia: a clinical update. The Journal of the American Osteopathic Association. 2013;113(9):680–9. 10.7556/jaoa.2013.034 . 24005088

[13] Clauw DJ. Fibromyalgia: a clinical review. JAMA: the journal of the American Medical Association. 2014;311(15):1547–55. 10.1001/jama.2014.3266 . 24737367

[14] Bernard AL, Prince A, Edsall P. Quality of life issues for fibromyalgia patients. Arthritis care and research: the official journal of the Arthritis Health Professions Association. 2000;13(1):42–50. . 11094925

[15] Burckhardt CS, Clark SR, Bennett RM. Fibromyalgia and quality of life: a comparative analysis. The Journal of rheumatology. 1993;20(3):475–9. . 8478854

[16] Bennett RM, Jones J, Turk DC, Russell IJ, Matallana L. An internet survey of 2,596 people with fibromyalgia. BMC musculoskeletal disorders. 2007;8:27 10.1186/1471-2474-8-27 17349056 PMC1829161

[17] Price DD, Staud R, Robinson ME, Mauderli AP, Cannon R, Vierck CJ. Enhanced temporal summation of second pain and its central modulation in fibromyalgia patients. Pain. 2002;99(1–2):49–59. . 12237183 10.1016/s0304-3959(02)00053-2

[18] Petzke F, Clauw DJ, Ambrose K, Khine A, Gracely RH. Increased pain sensitivity in fibromyalgia: effects of stimulus type and mode of presentation. Pain. 2003;105(3):403–13. . 14527701 10.1016/S0304-3959(03)00204-5

[19] Kosek E, Hansson P. Modulatory influence on somatosensory perception from vibration and heterotopic noxious conditioning stimulation (HNCS) in fibromyalgia patients and healthy subjects. Pain. 1997;70(1):41–51. . 9106808 10.1016/s0304-3959(96)03295-2

[20] Geisser ME, Casey KL, Brucksch CB, Ribbens CM, Appleton BB, Crofford LJ. Perception of noxious and innocuous heat stimulation among healthy women and women with fibromyalgia: association with mood, somatic focus, and catastrophizing. Pain. 2003;102(3):243–50. . 12670665 10.1016/S0304-3959(02)00417-7

[21] Kosek E, Ekholm J, Hansson P. Sensory dysfunction in fibromyalgia patients with implications for pathogenic mechanisms. Pain. 1996;68(2–3):375–83. . 9121827 10.1016/s0304-3959(96)03188-0

[22] Arroyo JF, Cohen ML. Abnormal responses to electrocutaneous stimulation in fibromyalgia. The Journal of rheumatology. 1993;20(11):1925–31. . 7848390

[23] Jensen KB, Kosek E, Petzke F, Carville S, Fransson P, Marcus H, et al Evidence of dysfunctional pain inhibition in Fibromyalgia reflected in rACC during provoked pain. Pain. 2009;144(1–2):95–100. 10.1016/j.pain.2009.03.018 . 19410366

[24] Jensen KB, Srinivasan P, Spaeth R, Tan Y, Kosek E, Petzke F, et al Overlapping structural and functional brain changes in patients with long-term exposure to fibromyalgia pain. Arthritis and rheumatism. 2013;65(12):3293–303. 10.1002/art.38170 23982850 PMC3984030

[25] Staud R, Nagel S, Robinson ME, Price DD. Enhanced central pain processing of fibromyalgia patients is maintained by muscle afferent input: a randomized, double-blind, placebo-controlled study. Pain. 2009;145(1–2):96–104. 10.1016/j.pain.2009.05.020 19540671 PMC2751583

[26] Bennett RM. Emerging concepts in the neurobiology of chronic pain: evidence of abnormal sensory processing in fibromyalgia. Mayo Clinic proceedings Mayo Clinic. 1999;74(4):385–98. 10.4065/74.4.385 . 10221469

[27] Uceyler N, Hauser W, Sommer C. Systematic review with meta-analysis: cytokines in fibromyalgia syndrome. BMC musculoskeletal disorders. 2011;12:245 10.1186/1471-2474-12-245 22034969 PMC3234198

[28] Uceyler N, Sommer C. Cytokine regulation in animal models of neuropathic pain and in human diseases. Neuroscience letters. 2008;437(3):194–8. 10.1016/j.neulet.2008.03.050 . 18403115

[29] Gur A, Karakoc M, Nas K, Remzi, Cevik, Denli A, et al Cytokines and depression in cases with fibromyalgia. The Journal of rheumatology. 2002;29(2):358–61. . 11838856

[30] Wallace DJ. Is there a role for cytokine based therapies in fibromyalgia. Current pharmaceutical design. 2006;12(1):17–22. . 16454720

[31] Kadetoff D, Lampa J, Westman M, Andersson M, Kosek E. Evidence of central inflammation in fibromyalgia-increased cerebrospinal fluid interleukin-8 levels. Journal of neuroimmunology. 2012;242(1–2):33–8. 10.1016/j.jneuroim.2011.10.013 . 22126705

[32] Sanada K, Diez MA, Valero MS, Perez-Yus MC, Demarzo MM, Garcia-Toro M, et al Effects of non-pharmacological interventions on inflammatory biomarker expression in patients with fibromyalgia: a systematic review. Arthritis research & therapy. 2015;17:272 10.1186/s13075-015-0789-9 26411586 PMC4584481

[33] Wang H, Buchner M, Moser MT, Daniel V, Schiltenwolf M. The role of IL-8 in patients with fibromyalgia: a prospective longitudinal study of 6 months. The Clinical journal of pain. 2009;25(1):1–4. 10.1097/AJP.0b013e31817e13a3 . 19158539

[34] Karshikoff B, Lekander M, Soop A, Lindstedt F, Ingvar M, Kosek E, et al Modality and sex differences in pain sensitivity during human endotoxemia. Brain Behav Immun. 2015;46:35–43. 10.1016/j.bbi.2014.11.014 . 25486090

[35] Imamura M, Targino RA, Hsing WT, Imamura S, Azevedo RS, Boas LS, et al Concentration of cytokines in patients with osteoarthritis of the knee and fibromyalgia. Clinical interventions in aging. 2014;9:939–44. 10.2147/CIA.S60330 24959074 PMC4061171

[36] Iannuccelli C, Di Franco M, Alessandri C, Guzzo MP, Croia C, Di Sabato F, et al Pathophysiology of fibromyalgia: a comparison with the tension-type headache, a localized pain syndrome. Annals of the New York Academy of Sciences. 2010;1193:78–83. 10.1111/j.1749-6632.2009.05365.x . 20398011

[37] Hernandez ME, Becerril E, Perez M, Leff P, Anton B, Estrada S, et al Proinflammatory cytokine levels in fibromyalgia patients are independent of body mass index. BMC research notes. 2010;3(1):156 10.1186/1756-0500-3-156 20525285 PMC2891797

[38] Wallace DJ, Linker-Israeli M, Hallegua D, Silverman S, Silver D, Weisman MH. Cytokines play an aetiopathogenetic role in fibromyalgia: a hypothesis and pilot study. Rheumatology. 2001;40(7):743–9. . 11477278 10.1093/rheumatology/40.7.743

[39] Bazzichi L, Rossi A, Massimetti G, Giannaccini G, Giuliano T, De Feo F, et al Cytokine patterns in fibromyalgia and their correlation with clinical manifestations. Clinical and experimental rheumatology. 2007;25(2):225–30. . 17543146

[40] Staud R. Cytokine and Immune System Abnormalities in Fibromyalgia and Other Central Sensitivity Syndromes. Curr Rheumatol Rev. 2015;11(2):109–15. . 26088214 10.2174/1573397111666150619094819

[41] Reichling DB, Green PG, Levine JD. The fundamental unit of pain is the cell. Pain. 2013;154 Suppl 1 10.1016/j.pain.2013.05.037 23711480 PMC3858489

[42] Gerdle B, Ghafouri B, Ernberg M, Larsson B. Chronic musculoskeletal pain: review of mechanisms and biochemical biomarkers as assessed by the microdialysis technique. Journal of pain research. 2014;7:313–26. 10.2147/JPR.S59144 24966693 PMC4062547

[43] Larsson B, Rosendal L, Kristiansen J, Sjogaard G, Sogaard K, Ghafouri B, et al Responses of algesic and metabolic substances to 8 h of repetitive manual work in myalgic human trapezius muscle. Pain. 2008;140(3):479–90. 10.1016/j.pain.2008.10.001 . 19006649

[44] Shah JP, Gilliams EA. Uncovering the biochemical milieu of myofascial trigger points using in vivo microdialysis: an application of muscle pain concepts to myofascial pain syndrome. Journal of bodywork and movement therapies. 2008;12(4):371–84. 10.1016/j.jbmt.2008.06.006 . 19083696

[45] Ghafouri B, Larsson BK, Sjors A, Leandersson P, Gerdle BU. Interstitial concentration of serotonin is increased in myalgic human trapezius muscle during rest, repetitive work and mental stress—an in vivo microdialysis study. Scandinavian journal of clinical and laboratory investigation. 2010;70(7):478–86. 10.3109/00365513.2010.511257 . 20712520

[46] Shah JP, Phillips TM, Danoff JV, Gerber LH. An in vivo microanalytical technique for measuring the local biochemical milieu of human skeletal muscle. Journal of applied physiology. 2005;99(5):1977–84. 10.1152/japplphysiol.00419.2005 . 16037403

[47] Rosendal L, Larsson B, Kristiansen J, Peolsson M, Sogaard K, Kjaer M, et al Increase in muscle nociceptive substances and anaerobic metabolism in patients with trapezius myalgia: microdialysis in rest and during exercise. Pain. 2004;112(3):324–34. 10.1016/j.pain.2004.09.017 . 15561388

[48] Ernberg M, Hedenberg-Magnusson B, Alstergren P, Kopp S. The level of serotonin in the superficial masseter muscle in relation to local pain and allodynia. Life sciences. 1999;65(3):313–25. . 10447217 10.1016/s0024-3205(99)00250-7

[49] Hedenberg-Magnusson B, Ernberg M, Alstergren P, Kopp S. Pain mediation by prostaglandin E2 and leukotriene B4 in the human masseter muscle. Acta odontologica Scandinavica. 2001;59(6):348–55. . 11831483 10.1080/000163501317153185

[50] Gerdle B, Soderberg K, Salvador Puigvert L, Rosendal L, Larsson B. Increased interstitial concentrations of pyruvate and lactate in the trapezius muscle of patients with fibromyalgia: a microdialysis study. Journal of rehabilitation medicine: official journal of the UEMS European Board of Physical and Rehabilitation Medicine. 2010;42(7):679–87. 10.2340/16501977-0581 . 20603699

[51] Gerdle B, Larsson B, Forsberg F, Ghafouri N, Karlsson L, Stensson N, et al Chronic widespread pain: increased glutamate and lactate concentrations in the trapezius muscle and plasma. The Clinical journal of pain. 2014;30(5):409–20. 10.1097/AJP.0b013e31829e9d2a . 23887335

[52] Gerdle B, Lemming D, Kristiansen J, Larsson B, Peolsson M, Rosendal L. Biochemical alterations in the trapezius muscle of patients with chronic whiplash associated disorders (WAD)—a microdialysis study. European journal of pain. 2008;12(1):82–93. 10.1016/j.ejpain.2007.03.009 . 17459742

[53] Rosendal L, Kristiansen J, Gerdle B, Sogaard K, Peolsson M, Kjaer M, et al Increased levels of interstitial potassium but normal levels of muscle IL-6 and LDH in patients with trapezius myalgia. Pain. 2005;119(1–3):201–9. 10.1016/j.pain.2005.09.026 . 16297553

[54] Littlejohn G. Neurogenic neuroinflammation in fibromyalgia and complex regional pain syndrome. Nature reviews Rheumatology. 2015;11(11):639–48. 10.1038/nrrheum.2015.100 . 26241184

[55] Bote ME, Garcia JJ, Hinchado MD, Ortega E. Fibromyalgia: anti-inflammatory and stress responses after acute moderate exercise. PloS one. 2013;8(9):e74524 10.1371/journal.pone.0074524 24023948 PMC3762808

[56] Larsson A, Palstam A, Lofgren M, Ernberg M, Bjersing J, Bileviciute-Ljungar I, et al Resistance exercise improves muscle strength, health status and pain intensity in fibromyalgia-a randomized controlled trial. Arthritis research & therapy. 2015;17:161 10.1186/s13075-015-0679-1 26084281 PMC4489359

[57] Borg G. Perceived exertion as an indicator of somatic stress. Scandinavian journal of rehabilitation medicine 1970;2(2):6. PubMed Central PMCID: PMC5523831. 5523831

[58] Bjelland I, Dahl AA, Haug TT, Neckelmann D. The validity of the Hospital Anxiety and Depression Scale. An updated literature review. Journal of psychosomatic research. 2002;52(2):69–77. . 11832252 10.1016/s0022-3999(01)00296-3

[59] Zigmond AS, Snaith RP. The hospital anxiety and depression scale. Acta psychiatrica Scandinavica. 1983;67(6):361–70. . 6880820 10.1111/j.1600-0447.1983.tb09716.x

[60] McHorney CA, Ware JE Jr, Raczek AE. The MOS 36-Item Short-Form Health Survey (SF-36): II. Psychometric and clinical tests of validity in measuring physical and mental health constructs. Medical care. 1993;31(3):247–63. . 8450681 10.1097/00005650-199303000-00006

[61] Sullivan M, Karlsson J. The Swedish SF-36 Health Survey III. Evaluation of criterion-based validity: results from normative population. Journal of clinical epidemiology. 1998;51(11):1105–13. . 9817128 10.1016/s0895-4356(98)00102-4

[62] Persson LO, Karlsson J, Bengtsson C, Steen B, Sullivan M. The Swedish SF-36 Health Survey II. Evaluation of clinical validity: results from population studies of elderly and women in Gothenborg. Journal of clinical epidemiology. 1998;51(11):1095–103. . 9817127 10.1016/s0895-4356(98)00101-2

[63] Jansen JJ, Szymanska E, Hoefsloot HC, Jacobs DM, Strassburg K, Smilde AK. Between Metabolite Relationships: an essential aspect of metabolic change. Metabolomics. 2012;8(3):422–32. Epub 2012/06/05. 10.1007/s11306-011-0316-1 316 [pii]. 22661919 PMC3351608

[64] Pohjanen E, Thysell E, Jonsson P, Eklund C, Silfver A, Carlsson IB, et al A multivariate screening strategy for investigating metabolic effects of strenuous physical exercise in human serum. J Proteome Res. 2007;6(6):2113–20. Epub 2007/04/13. 10.1021/pr070007g . 17428078

[65] Eriksson L, Johansson E, Kettaneh-Wold N, Trygg J, Wikström C, Wold S. Multi- and Megavariate Data analysis; part I and II. 2 ed. Umeå: Umetrics AB; 2006.

[66] Tan AR, Dowlati A, Jones SF, Infante JR, Nishioka J, Fang L, et al Phase I study of pazopanib in combination with weekly paclitaxel in patients with advanced solid tumors. The oncologist. 2010;15(12):1253–61. 10.1634/theoncologist.2010-0095 21147873 PMC3227920

[67] Dowlati Y, Herrmann N, Swardfager W, Thomson S, Oh PI, Van Uum S, et al Relationship between hair cortisol concentrations and depressive symptoms in patients with coronary artery disease. Neuropsychiatric disease and treatment. 2010;6:393–400. 20856603 PMC2938288

[68] Gerdle B, Hilgenfeldt U, Larsson B, Kristiansen J, Sogaard K, Rosendal L. Bradykinin and kallidin levels in the trapezius muscle in patients with work-related trapezius myalgia, in patients with whiplash associated pain, and in healthy controls—A microdialysis study of women. Pain. 2008;139(3):578–87. 10.1016/j.pain.2008.06.012 . 18657364

[69] Thyberg I, Skogh T, Hass UA, Gerdle B. Recent-onset rheumatoid arthritis: a 1-year observational study of correlations between health-related quality of life and clinical/laboratory data. Journal of rehabilitation medicine: official journal of the UEMS European Board of Physical and Rehabilitation Medicine. 2005;37(3):159–65. 10.1080/16501970410023344 . 16040473

[70] Sandberg M, Zhang Q, Styf J, Gerdle B, Lindberg LG. Non-invasive monitoring of muscle blood perfusion by photoplethysmography: evaluation of a new application. Acta physiologica Scandinavica. 2005;183(4):335–43. 10.1111/j.1365-201X.2005.01412.x . 15799770

[71] Larsson B, Kadi F, Lindvall B, Gerdle B. Surface electromyography and peak torque of repetitive maximum isokinetic plantar flexions in relation to aspects of muscle morphology. Journal of electromyography and kinesiology: official journal of the International Society of Electrophysiological Kinesiology. 2006;16(3):281–90. 10.1016/j.jelekin.2005.07.009 . 16129622

[72] Jensen-Waern M, Persson SG, Nordengrahn A, Merza M, Fossum C. Temporary suppression of cell-mediated immunity in standardbred horses with decreased athletic capacity. Acta Vet Scand. 1998;39(1):25–33. . 9592943 10.1186/BF03547804 PMC8050687

[73] Bergquist A, Glaumann H, Persson B, Broome U. Risk factors and clinical presentation of hepatobiliary carcinoma in patients with primary sclerosing cholangitis: a case-control study. Hepatology. 1998;27(2):311–6. 10.1002/hep.510270201 . 9462625

[74] Li YP, Niu A, Wen Y. Regulation of myogenic activation of p38 MAPK by TACE-mediated TNFalpha release. Front Cell Dev Biol. 2014;2:21 10.3389/fcell.2014.00021 25364728 PMC4207040

[75] Dawson A, List T, Ernberg M, Svensson P. Assessment of proprioceptive allodynia after tooth-clenching exercises. Journal of orofacial pain. 2012;26(1):39–48. . 22292139

[76] Ernberg MM, Alstergren PJ. Microdialysis of neuropeptide Y in human muscle tissue. Journal of neuroscience methods. 2004;132(2):185–90. . 14706716 10.1016/j.jneumeth.2003.09.009

[77] Uceyler N, Valenza R, Stock M, Schedel R, Sprotte G, Sommer C. Reduced levels of antiinflammatory cytokines in patients with chronic widespread pain. Arthritis and rheumatism. 2006;54(8):2656–64. 10.1002/art.22026 . 16871547

[78] Kadetoff D, Kosek E. The effects of static muscular contraction on blood pressure, heart rate, pain ratings and pressure pain thresholds in healthy individuals and patients with fibromyalgia. European journal of pain. 2007;11(1):39–47. 10.1016/j.ejpain.2005.12.013 . 16480906

[79] Kunos CA, Waggoner S, von Gruenigen V, Eldermire E, Pink J, Dowlati A, et al Phase I trial of pelvic radiation, weekly cisplatin, and 3-aminopyridine-2-carboxaldehyde thiosemicarbazone (3-AP, NSC #663249) for locally advanced cervical cancer. Clinical cancer research: an official journal of the American Association for Cancer Research. 2010;16(4):1298–306. 10.1158/1078-0432.CCR-09-2469 20145183 PMC2822897

[80] Sinyor M, Levitt AJ, Cheung AH, Schaffer A, Kiss A, Dowlati Y, et al Does inclusion of a placebo arm influence response to active antidepressant treatment in randomized controlled trials? Results from pooled and meta-analyses. The Journal of clinical psychiatry. 2010;71(3):270–9. 10.4088/JCP.08r04516blu . 20122371

[81] Uceyler N, Topuzoglu T, Schiesser P, Hahnenkamp S, Sommer C. IL-4 deficiency is associated with mechanical hypersensitivity in mice. PloS one. 2011;6(12):e28205 10.1371/journal.pone.0028205 22164245 PMC3229527

[82] McIver KL, Evans C, Kraus RM, Ispas L, Sciotti VM, Hickner RC. NO-mediated alterations in skeletal muscle nutritive blood flow and lactate metabolism in fibromyalgia. Pain. 2006;120(1–2):161–9. 10.1016/j.pain.2005.10.032 . 16376018

[83] Berg U, Gustafsson T, Sundberg CJ, Carlsson-Skwirut C, Hall K, Jakeman P, et al Local changes in the insulin-like growth factor system in human skeletal muscle assessed by microdialysis and arterio-venous differences technique. Growth hormone & IGF research: official journal of the Growth Hormone Research Society and the International IGF Research Society. 2006;16(4):217–23. 10.1016/j.ghir.2006.05.004 . 16904923

[84] Clough GF. Microdialysis of large molecules. The AAPS journal. 2005;7(3):E686–92. 10.1208/aapsj070369 16353945 PMC2751271

[85] Carson BP, McCormack WG, Conway C, Cooke J, Saunders J, O'Connor WT, et al An in vivo microdialysis characterization of the transient changes in the interstitial dialysate concentration of metabolites and cytokines in human skeletal muscle in response to insertion of a microdialysis probe. Cytokine. 2015;71(2):327–33. 10.1016/j.cyto.2014.10.022 . 25528289

[86] Torgrimson-Ojerio B, Ross RL, Dieckmann NF, Avery S, Bennett RM, Jones KD, et al Preliminary evidence of a blunted anti-inflammatory response to exhaustive exercise in fibromyalgia. Journal of neuroimmunology. 2014;277(1–2):160–7. 10.1016/j.jneuroim.2014.10.003 25457842 PMC4314393

[87] Dowlati Y, Herrmann N, Swardfager W, Liu H, Sham L, Reim EK, et al A meta-analysis of cytokines in major depression. Biological psychiatry. 2010;67(5):446–57. 10.1016/j.biopsych.2009.09.033 . 20015486

[88] Kim YK, Na KS, Shin KH, Jung HY, Choi SH, Kim JB. Cytokine imbalance in the pathophysiology of major depressive disorder. Progress in neuro-psychopharmacology & biological psychiatry. 2007;31(5):1044–53. 10.1016/j.pnpbp.2007.03.004 . 17433516

